# Recent Advances in Two-Dimensional Bismuth Oxysulfide

**DOI:** 10.3390/ijms27156607

**Published:** 2026-07-24

**Authors:** Donghun Lee

**Affiliations:** Department of Chemistry, Kookmin University, Seoul 02707, Republic of Korea; donghunlee@kookmin.ac.kr

**Keywords:** Bi_2_O_2_S, two-dimensional materials, field-effect transistors

## Abstract

Two-dimensional (2D) semiconductors have attracted significant attention for their potential in low-power and high-performance electronic applications. Among these, bismuth oxysulfide (Bi_2_O_2_S) has recently emerged as a candidate owing to its wide bandgap, low effective electron mass, and environmental stability. This review summarizes the synthesis strategies and device applications of 2D Bi_2_O_2_S. It systematically analyzes recent advancements in synthesis methodologies, ranging from scalable solution-based synthesis to back-end-of-line compatible, low-temperature metal–organic chemical vapor deposition. Furthermore, defect engineering, particularly through oxygen-vacancy control and doping, is discussed as a strategy to modulate the electronic band structure, enhance broadband nonlinear optical properties, and accelerate the photocarrier relaxation kinetics of 2D Bi_2_O_2_S. These tunable characteristics have enabled 2D Bi_2_O_2_S to be employed in electronic and optoelectronic devices. The review, therefore, discusses recent developments in device applications of Bi_2_O_2_S, including field-effect transistors, broadband photodetectors, and flexible photoelectrochemical sensors. Finally, the remaining challenges, such as wafer-scale single-crystal growth and reliable p-type doping, and future research directions are discussed for integrating Bi_2_O_2_S into three-dimensional integrated circuits and neuromorphic computing.

## 1. Introduction

**Figure 1:**
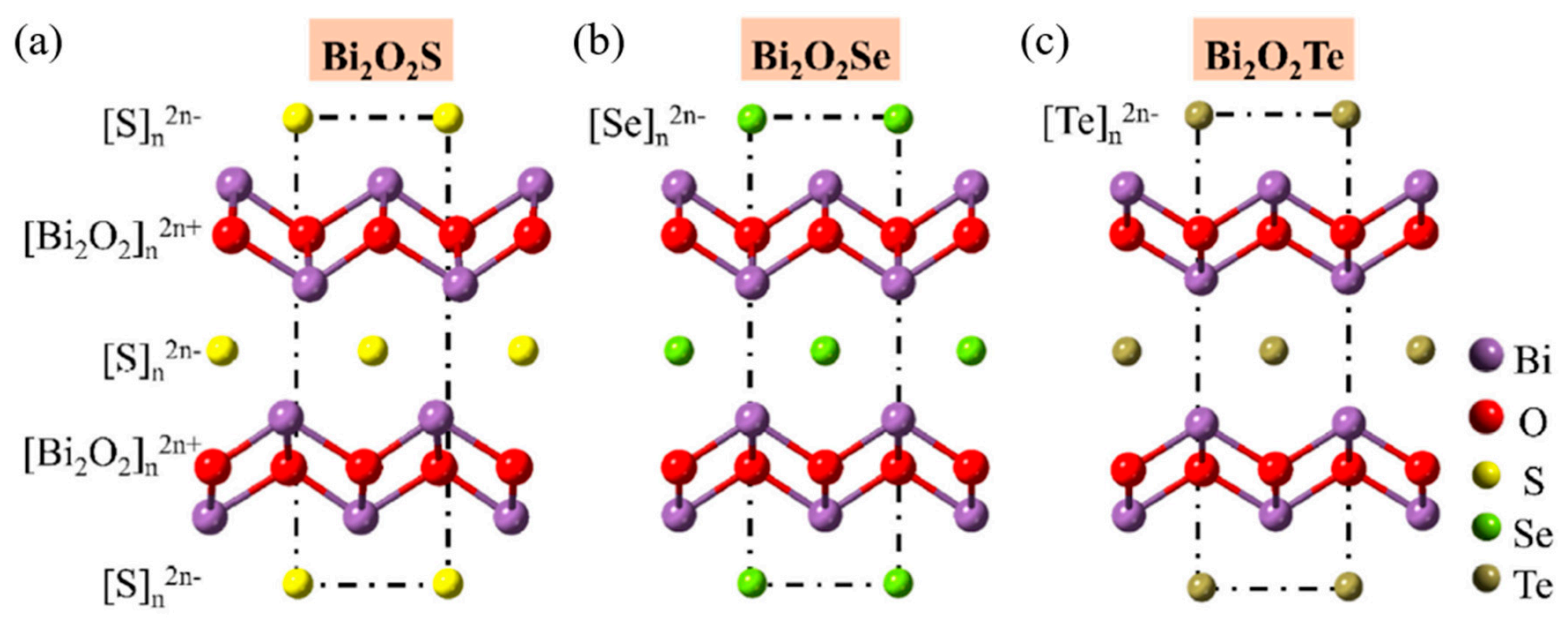
(**a**–**c**) Schematic representation of the atomic configurations for (**a**) Bi_2_O_2_S, (**b**) Bi_2_O_2_Se, and (**c**) Bi_2_O_2_Te. Reproduced with permission [12].

The emergence of two-dimensional (2D) materials has led to the development of next-generation electronic and optoelectronic devices [1,2,3,4,5,6]. However, a fundamental trade-off among bandgap size, carrier transport, and environmental stability remains a key challenge for atomically thin semiconductors. For example, graphene exhibits exceptional carrier mobility but lacks an intrinsic bandgap, whereas many transition-metal dichalcogenides (TMDs) have finite bandgaps but generally exhibit limited room-temperature mobility [7,8,9]. Although black phosphorus bridges the gap between transport and electrostatic switching, its rapid degradation under ambient conditions remains a practical limitation [10,11]. Bismuth oxychalcogenide (Bi_2_O_2_X, where X = S, Se, or Te) has emerged as a new type of layered semiconductor capable of overcoming these limitations [12,13,14]. Unlike conventional van der Waals crystals, these materials consist of positively charged [Bi_2_O_2_]^2+^ slabs and X^2−^ anion layers that are stacked via relatively weak electrostatic interactions. This results in an ionic layered structure with high surface stability and thickness-dependent electronic properties (Figure 1). Initial studies on Bi_2_O_2_X focused mainly on ultrathin bismuth oxyselenide (Bi_2_O_2_Se) owing to its excellent electronic properties, such as a Hall mobility of 28,900 cm^2^ V^−1^ s^−1^ at 1.9 K and a near-ideal subthreshold swing of ~65 mV dec^−1^ [15,16,17]. However, the narrow bandgap of Bi_2_O_2_Se (~0.8 eV) increases off-state leakage currents, making it difficult to use in low-power switching applications. Therefore, bismuth oxysulfide (Bi_2_O_2_S) has attracted growing attention as an alternative channel material owing to its wider bandgap [18,19,20,21].

Bi_2_O_2_S has an orthorhombic crystal structure belonging to the *Pnnm* space group, whereas Bi_2_O_2_Se exhibits a highly symmetric tetragonal structure (*I4/mmm*) [13,22]. With a reported bandgap ranging from 1.1 to 1.5 eV, Bi_2_O_2_S-based photodetectors exhibit spectral responses from the ultraviolet (UV) to the near-infrared (NIR) regime [23,24]. Orthorhombic distortion is of particular interest because it breaks local centrosymmetry and induces polar responses in 2D Bi_2_O_2_S [25,26]. Ultrathin Bi_2_O_2_S nanosheets particularly exhibit room-temperature ferroelectricity, whereas epitaxial films show antiferroelectric order, indicating that the polar ground state is sensitive to thickness and structural alignment. This unique structural asymmetry and ferroelectric behavior of Bi_2_O_2_S make it a candidate for non-volatile memory and energy-efficient neuromorphic computing.

## 2. Crystal Structure and Electrical Properties

### 2.1. Crystal and Electronic Band Structure

**Figure 2:**
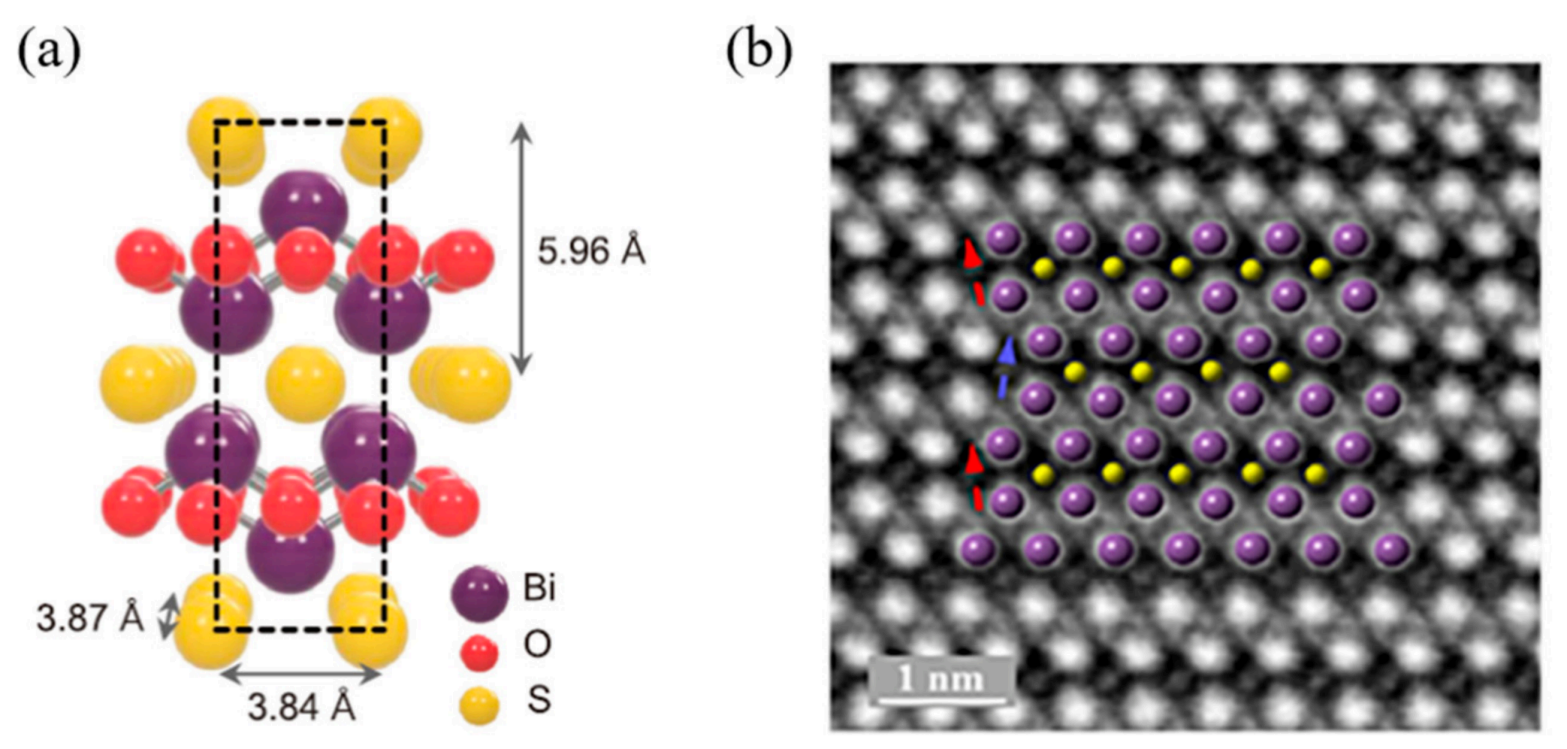
(**a**) Schematic representation of the orthorhombic crystal structure of Bi_2_O_2_S, illustrating the lattice parameters across the three principal crystallographic axes. Reproduced with permission [23]. (**b**) Cross-sectional high-angle annular dark-field image of a Bi_2_O_2_S film, where the arrows indicate the direction of structural distortions. Reproduced with permission [25].

Bi_2_O_2_S is characterized as an ionic layered semiconductor rather than a conventional van der Waals material. It consists of [Bi_2_O_2_]^2+^ cation and S^2−^ anion layers stacked alternately along the out-of-plane direction [23,25]. In contrast to tetragonal Bi_2_O_2_Se, Bi_2_O_2_S exhibits an orthorhombic structure (a ≈ 3.84 Å, b ≈ 3.87 Å, and c ≈ 11.92 Å) (Figure 2). The resulting in-plane anisotropy arises from the relative displacement of adjacent [Bi_2_O_2_] layers.

Generally, Bi_2_O_2_S is classified as an indirect bandgap semiconductor. First-principles calculations predict that as the thickness of Bi_2_O_2_S decreases from bulk toward monolayer, the bandgap widens owing to weakened interlayer coupling and quantum confinement [26,27]. However, the monolayer value depends on the surface termination. In calculations for Bi_2_O_2_S with a pristine surface, the bandgap is predicted to be as large as 2.6 eV, whereas a hydrogen-passivated monolayer exhibits 1.49 and 1.92 eV [26,28,29]. Furthermore, Bi_2_O_2_S exhibits a low effective electron mass of approximately 0.15 *m*_0_ along the Γ–X direction, which enables high carrier mobility of 16,447 cm^2^ V^−1^ s^−1^. However, the experimentally measured mobility is generally lower than the calculated values because defect density, interfacial roughness, contact resistance, and the surrounding dielectric environment suppress carrier transport behavior. Compared to technologically mature 2D semiconductors such as MoS_2_, research on Bi_2_O_2_S is still in its early stages in terms of wafer-scale synthesis, device integration, and long-term reliability. Consequently, significant technological breakthroughs are required for its practical application.

### 2.2. Ferroelectric Characteristics

In its bulk form, Bi_2_O_2_S crystallizes in the centrosymmetric orthorhombic space group (*Pnnm*), which theoretically forbids spontaneous macroscopic polarization. However, in its ultrathin form, the material undergoes structural distortion [26]. Specifically, the localized atomic displacement of bismuth and oxygen atoms within the [Bi_2_O_2_]^2+^ layers distorts the *D*_2h_ rhombic network into a lower-symmetry *C*_2v_ configuration. This structural distortion breaks the local inversion symmetry, resulting in room-temperature 2D ferroelectricity. The polarization dynamics of Bi_2_O_2_S are, therefore, closely related to its interlayer coupling. Density functional theory (DFT) simulations demonstrate that the dipole moments of adjacent [Bi_2_O_2_]^2+^ slabs align in opposite directions, even though the individual monolayer is ferroelectric. This antiferroelectric interaction results in distinct layer-dependent behavior. Nanosheets with an odd number of layers display uncompensated residual dipole moments that yield a net 2D ferroelectricity, whereas those with an even number of layers exhibit zero net macroscopic polarization due to antiferroelectric compensation.

**Figure 3:**
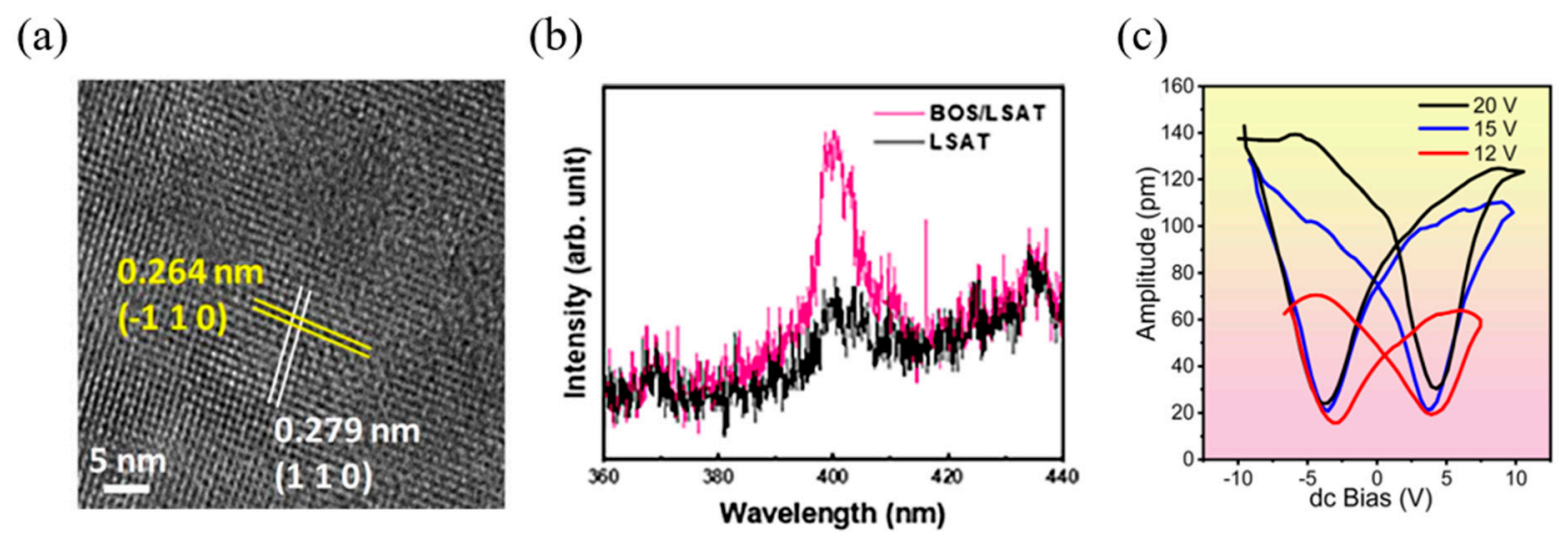
(**a**) HRTEM image viewed along the [001] zone axis, illustrating the discrepancy in interplanar spacings between the (110) and (−110) crystallographic directions. Reproduced with permission [26]. (**b**) SHG intensity spectra comparing an epitaxial Bi_2_O_2_S thin film and a bare (LaAlO_3_)_0.3_(Sr_2_TaAlO_6_)_0.7_ (LSAT) substrate. Reproduced with permission [25]. (**c**) Piezoresponse amplitude loops of Bi_2_O_2_S nanosheets acquired under different applied DC biases. Reproduced with permission [26].

Confirming the emerging polar states in Bi_2_O_2_S demands a strict distinction between structural non-centrosymmetry and true, switchable ferroelectric polarization, as the indirect signals obtained from various characterization techniques cannot equally support the existence of polar states. For example, high-resolution transmission electron microscopy (HRTEM) revealed unequal spacings between nominally symmetry-equivalent (110) and (−110) planes, directly visualizing the local symmetry breakdown (Figure 3a) [26]. However, while such atomic displacements confirm static non-centrosymmetry, they cannot verify the dynamic reversibility of the dipole moments under an external electric field. In addition, second-harmonic generation (SHG) measurements confirmed the presence of macroscopic inversion symmetry breaking (Figure 3b) [25]. However, SHG alone cannot verify the deterministic reversibility of these polar states, as the measured responses can be influenced by localized surface/interfacial defects or uncompensated edge electronic states [30,31]. These structural and optical measurements should therefore be regarded as indirect indicators of non-centrosymmetric polar lattices rather than direct evidence of switchable ferroelectric properties. Switchable macroscopic polarization in bulk materials is traditionally quantified via direct polarization-electric field hysteresis loops. However, such piezoelectric force microscopy (PFM) signals in ultrathin materials should be interpreted with appropriate caution, as electrostatic effects, charge injection, ionic motion, or electrochemical strain can also generate ferroelectric-like hysteresis [32]. To eliminate these non-ferroelectric artifacts, the switching spectroscopy PFM (SS-PFM) method has been developed [33]. In SS-PFM mode, the bias is applied as a series of short DC voltage pulses, and the local electromechanical response is recorded during the “off-state” between pulses. This off-state detection method minimizes the influence of transient electrostatic effects, enabling accurate evaluation of the intrinsic remanent polarization. Pathak et al. therefore conducted SS-PFM measurements, which clearly exhibited butterfly-shaped amplitude loops, as shown in Figure 3c [26]. Recently synthesized Bi_2_O_2_S thin films are predominantly polycrystalline, and the influence of grain boundaries on carrier transport, polarization behavior, and device uniformity remains unexplored. In such polycrystalline thin films, the presence of high-density grain boundaries and structural defects can shield the interior ferroelectric dipoles from external switching fields. Consequently, the macroscopic ferroelectric switching required for non-volatile memories is substantially suppressed or degraded in polycrystalline Bi_2_O_2_S devices. Therefore, the integration of these materials into practical devices will depend on future progress in single-crystal thin-film growth and interface engineering.

## 3. Synthesis of 2D Bi_2_O_2_S Nanosheets

### 3.1. Solution-Phase Synthesis and Morphological Control

The synthesis of high-quality 2D crystals often faces challenges due to specific requirements, such as high-vacuum and high-temperature conditions [34,35,36]. Alternatively, solution-based strategies, including mild wet-chemical and hydrothermal methods, provide distinct advantages for the mass production of Bi_2_O_2_S nanostructures owing to their scalability, cost-effectiveness, and low-temperature processing [24,27,37,38]. Furthermore, the morphology, dimensions, and defect density of Bi_2_O_2_S can be precisely controlled by tuning the precursor chemical composition, regulating the pH of the reaction solution, or introducing organic templating agents.

**Figure 4:**
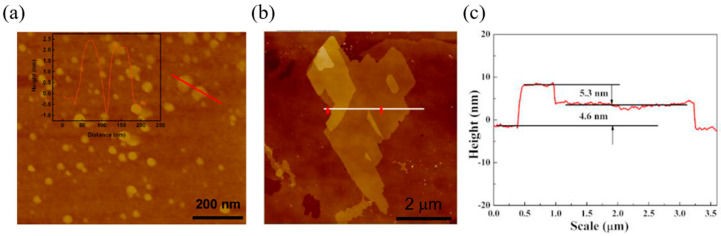
(**a**) AFM topographic image of Bi_2_O_2_S nanosheets prepared via a room-temperature wet-chemical synthesis route. The inset displays the extracted height profile measured along the solid red line. Reproduced with permission [37]. (**b**) AFM image of a Bi_2_O_2_S nanosheet grown using the hydrothermal method. (**c**) Corresponding height profile measured along the white line indicated in (**b**). Reproduced with permission [38].

Recently, the facile preparation of freestanding ultrathin Bi_2_O_2_S nanosheets has been successfully demonstrated via a room-temperature wet-chemical approach [26,37]. This method does not require high-pressure autoclaves or highly toxic reducing agents, such as hydrazine hydrate, commonly used in conventional hydrothermal synthesis methods. The process is typically initiated by hydrolyzing bismuth nitrate pentahydrate (Bi(NO_3_)_3_⋅5H_2_O) in a strongly alkaline aqueous environment to form BiONO_3_ intermediates. Subsequently, the decomposition of thiourea (CS(NH_2_)_2_) gradually releases sulfide ions (S^2−^), which react with the BiONO_3_ intermediates. The spontaneous crystallization of Bi_2_O_2_S is fundamentally driven by the electrostatic co-assembly of the [Bi_2_O_2_]^2+^ cationic frameworks and the S^2−^ anionic planes. This room-temperature solution process yields single-crystalline nanosheets with a thickness of 2 nm and lateral dimensions of 20–40 nm (Figure 4a).

While mild wet-chemical reactions are excellent for producing ultrathin Bi_2_O_2_S, hydrothermal synthesis provides superior kinetic control over the lateral size and structural assembly of the crystals by using appropriate organic surfactants [38,39]. For example, replacing conventional bismuth nitrate precursors with ammonium bismuth citrate (C_6_H_13_BiN_2_O_7_⋅H_2_O) induces a highly effective template-guided anisotropic growth pathway [38]. During the hydrothermal reaction, organic citrate anions ([(NH_3_)_2_C_6_H_7_O_7_]^3−^) strongly bind to Bi^3+^ ions on the Bi-rich (001) basal planes. This selective surface passivation hinders the diffusion of adatoms along the c-axis, causing the crystal to expand laterally and thus yielding well-crystallized Bi_2_O_2_S nanosheets with large lateral dimensions exceeding 2.0 μm (Figure 4b,c).

Beyond individual 2D flake synthesis, finely tuning hydrothermal synthesis parameters can yield complex three-dimensional (3D) hierarchical structures. For example, Rong et al. successfully fabricated 3D Bi_2_O_2_S microflowers using ethylene glycol (EG) as a soft template and water as a solvent [40]. The initially nucleated 2D ultrathin nanosheets, with a thickness of 12–16 nm, serve as the primary building blocks in this morphological evolution. These constituent nanosheets, driven by the hydrogen bonding cross-linking network of EG, become oriented and intertwined with each other, ultimately forming a well-defined spherical flower-shaped microstructure.

A particularly notable feature of the Bi–O–S material system is its capacity for distinct functional phase transitions under thermal treatment. Specifically, hydrothermally synthesized Bi_2_O_2_S has been shown to decompose after annealing at 500 °C in an evacuated quartz tube, yielding tetragonal Bi_4_O_4_S_3_ together with Bi and Bi_2_O_3_ according to the following reaction: 3Bi_2_O_2_S → Bi_4_O_4_S_3_ + 2/3Bi + 2/3Bi_2_O_3_ [27]. The resulting Bi_4_O_4_S_3_ exhibits a superconducting transition at 4.6 K.

### 3.2. Vapor-Phase Growth of Bi_2_O_2_S

Although solution-phase synthesis yields individual 2D Bi_2_O_2_S flakes, controllable vapor-phase synthesis techniques remain essential for fabricating large-area single crystals for integrated circuit devices [41,42,43,44]. While Bi_2_O_2_Se has been synthesized via powder-based chemical vapor deposition (CVD), Bi_2_O_2_S suffers from synthetic bottlenecks under this route [45,46,47,48,49]. This difficulty originates from the low vapor pressures of conventional solid precursors, including Bi_2_O_3_ and Bi_2_S_3_, thereby requiring a high sublimation temperature exceeding 700 °C [23]. According to the ternary phase diagrams, at these elevated temperatures, the formation of secondary impurity phases, such as Bi_2_OS_2_ and Bi_14_SO_24_, is thermodynamically favored over the desired Bi_2_O_2_S. Consequently, it is difficult to obtain phase-pure Bi_2_O_2_S using conventional powder-based CVD methods.

**Figure 5:**
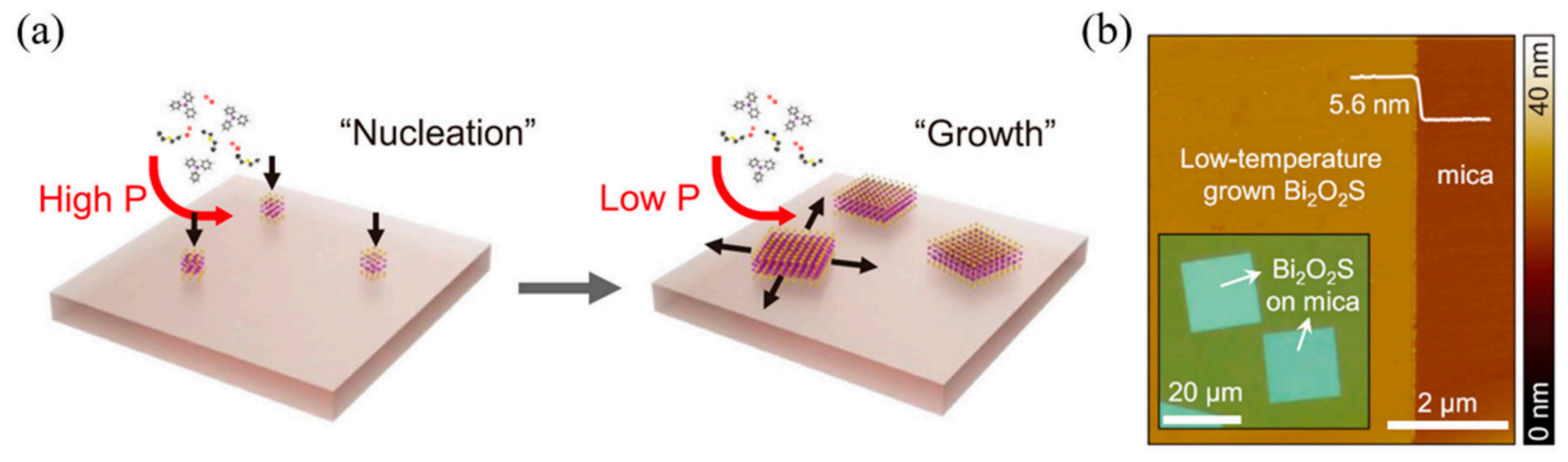
(**a**) Schematic of the pressure-dependent two-step growth mechanism of Bi_2_O_2_S via MOCVD. (**b**) AFM topography and the corresponding optical micrograph (inset) of a Bi_2_O_2_S nanoplate. Reproduced with permission [23].

To address these fundamental thermodynamic bottlenecks, a metal–organic CVD (MOCVD) approach utilizing highly volatile organic precursors has been developed to lower the reaction temperature for Bi_2_O_2_S [23]. In this study, Bi_2_O_2_S was synthesized on a mica substrate at 400–430 °C using triphenylbismuth (Bi(Ph)_3_) and diethyl sulfide with a precisely controlled oxygen supply. This substantial reduction in the thermal budget suppresses the thermodynamic driving force for secondary-phase formation, ensuring the growth of phase-pure Bi_2_O_2_S. This low-temperature Bi_2_O_2_S synthesis is enabled through a two-step kinetic control via chamber-pressure modulation (Figure 5). During the initial nucleation step, a relatively high chamber pressure (~61.5 Torr) is sustained to enhance gas-phase collision rates, thereby driving supersaturation and promoting a high density of nucleation sites on substrates. Subsequently, the chamber pressure is reduced below 43.5 Torr to promote the lateral growth of nuclei while suppressing excessive out-of-plane growth. This method yields highly crystalline 2D nanoplates with large lateral dimensions of tens of micrometers and nanometer-scale thicknesses. This approach provides a reliable synthetic route for producing device-grade Bi_2_O_2_S. Furthermore, direct synthesis of this high-mobility 2D semiconductor with a bandgap of 1.12–1.2 eV below 400 °C is highly compatible with the stringent thermal budget required for back-end-of-line (BEOL) integration [44,45,46].

### 3.3. Epitaxial Growth of Bi_2_O_2_S Thin Films

Although CVD and solution-based methodologies are effective for synthesizing isolated 2D flakes, random grain boundaries and structural defects remain critical bottlenecks for wafer-scale monolithic integration [50,51,52]. In this regard, heteroepitaxy has emerged as a key strategy to achieve high-quality, large-area Bi_2_O_2_S thin films with uniformly aligned crystallographic orientations.

**Figure 6:**
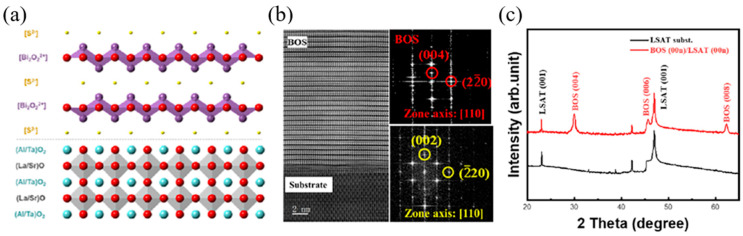
(**a**) Schematic illustration of the structural configuration of the Bi_2_O_2_S/LSAT heteroepitaxial structure. (**b**) Cross-sectional STEM image and corresponding fast Fourier-transform patterns of a PLD-grown Bi_2_O_2_S film on an LSAT substrate. (**c**) X-ray diffraction patterns of the epitaxial Bi_2_O_2_S film grown on LSAT. Reproduced with permission [25].

Recent advancements have demonstrated the epitaxial growth of Bi_2_O_2_S films via pulsed laser deposition (PLD) by utilizing substrates with highly compatible lattice constants (Figure 6) [25]. For example, cubic LSAT substrates (a = b = c = 0.384 nm) have been employed to guide the heteroepitaxial growth of Bi_2_O_2_S (a = b = 0.387 nm, c = 1.192 nm). This small lattice mismatch suppresses interfacial strain and establishes a well-defined epitaxial relationship of (002)BOS//(001)LSAT at the heterogeneous interface. X-ray diffraction and atomic-scale scanning transmission electron microscopy (STEM) confirm that these epitaxial films exhibit atomically sharp interfaces without secondary impurity phases.

Table 1 provides a summary of the various synthesis methods for Bi_2_O_2_S.

## 4. Defect Engineering and Property Modulation

### 4.1. Oxygen-Vacancy Engineering in Bi_2_O_2_S

**Figure 7:**
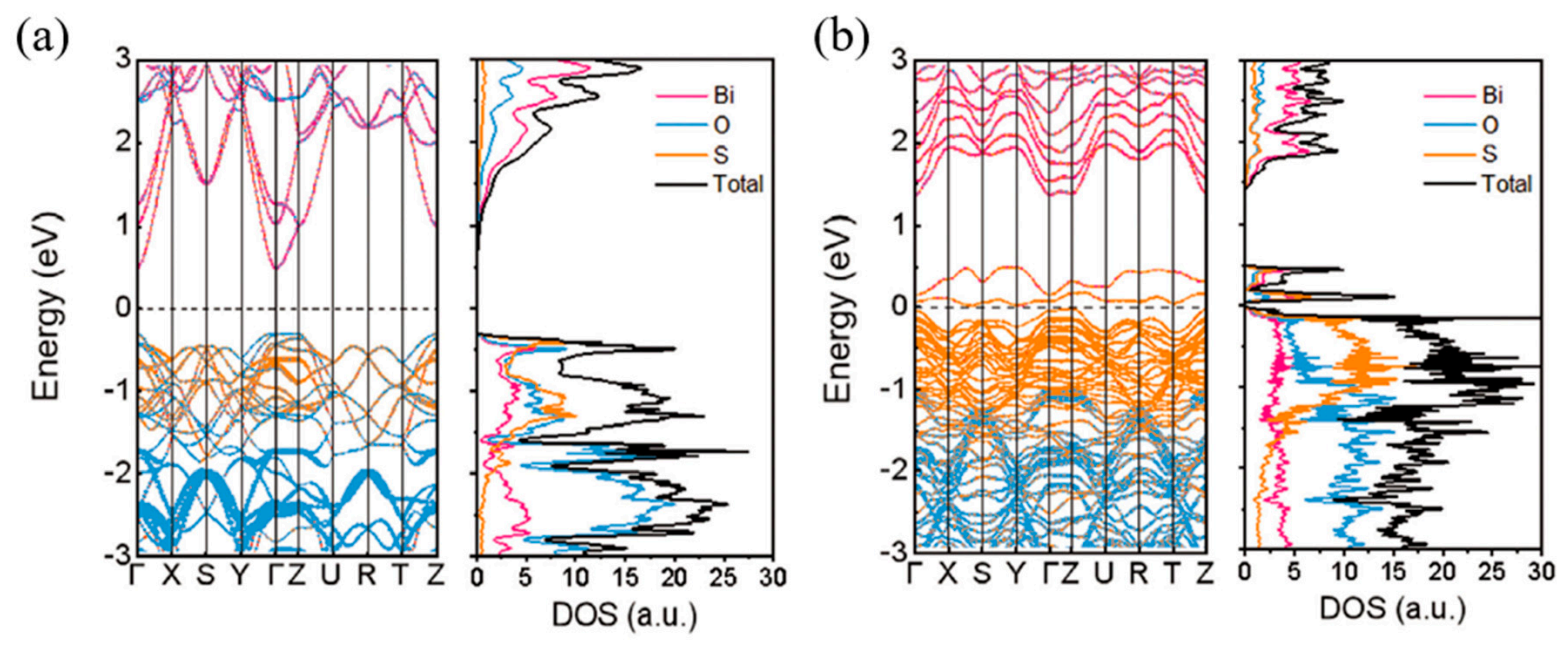
(**a**,**b**) DFT-simulated band structures (left column) and density of states (right column) for Bi_2_O_2_S lattices containing (**a**) 0% and (**b**) 12.5% OVs. Reproduced with permission [24].

In low-dimensional semiconductors, structural defects are no longer viewed only as detrimental factors but as effective means to modulate electrical properties. Recently, the selective engineering of oxygen vacancies (OVs) within the [Bi_2_O_2_]^2+^ cationic frameworks of the 2D Bi_2_O_2_S system has emerged as an effective route to modulate its electronic band structure and optical properties [24]. The intentional introduction of OVs generates defect-induced sub-bands within the bandgap between the conduction-band edge and the Fermi level (Figure 7). For example, increasing the OV concentration to about 35% narrows the effective optical bandgap from its intrinsic value of 1.21 eV down to 0.81 eV. Beyond modulating the optical bandgap, OVs affect the transient dynamics of photogenerated charge carriers. Femtosecond pump–probe measurements reveal a biexponential decay profile, with both lifetime components decreasing with increasing vacancy density. Specifically, the fast component decreases from ~2.40 ps for low OV-density samples to ~0.48 ps for high OV-density samples, while the slow component decreases from ~30.76 ps to ~12.71 ps. The fast relaxation is attributed to charge trapping by defect-related local states, whereas the slow relaxation originates from defect-assisted nonradiative recombination.

### 4.2. Anion-Site Substitution and Defect Chemistry

Beyond vacancy control, chalcogen-site substitution provides another route to modify the electronic structure of Bi_2_O_2_S, where sulfur is replaced by selenium [24]. Since S and Se share similar chemical properties, introduction of Se into the S layer modulates the electronic band structure while preserving the fundamental orthorhombic lattice. In Se-substituted Bi_2_O_2_S nanosheets prepared via controlled hydrothermal synthesis, this substitution induces a measurable lattice expansion arising from the ionic radius difference between S^2−^ (1.84 Å) and Se^2−^ (1.98 Å). HRTEM measurements revealed that the (101) interplanar spacing expands from 0.27 nm in pristine samples to 0.28 nm in highly alloyed compositions (e.g., 30% Se). This incorporation of Se substituents substantially reconstructs the electronic band alignment. As the Se substitution ratio increases from 20% to 50%, the optical bandgap decreases from 1.08 eV to 0.29 eV. Consequently, Se-substituted Bi_2_O_2_S exhibits broadband absorption characteristics extending from the visible spectrum to 1500 nm.

While the bandgap of Bi_2_O_2_S can be tuned through defect engineering, a primary concern is whether OVs or Se substitution preserves the pristine Bi_2_O_2_S phase. Specifically, X-ray diffraction, Raman spectroscopy, and selected-area electron diffraction measurements confirmed that Bi_2_O_2_S nanosheets with high OV concentrations (up to 35%) and Se substitution (up to 50%) exhibited a single-crystalline orthorhombic phase. While these structural defects induce bandgap narrowing, they inevitably introduce localized trap states within the bandgap that alter photocarrier dynamics. These defects serve as efficient trapping and recombination centers, accelerating photocarrier relaxation to the sub-picosecond regime (~0.48 ps for 35% OV and ~0.14 ps for 50% Se). Such defect states are advantageous for ultrafast nonlinear optical applications, such as mode-locked pulsed lasers, due to this rapid carrier recovery. On the other hand, this defect-assisted recombination and trapping phenomenon shortens the lifetime of free charge carriers and inhibits efficient charge separation, thereby reducing the extraction efficiency of photodetection and photovoltaic devices.

## 5. High-Performance Electronic and Optoelectronic Applications

### 5.1. Field-Effect Transistors (FETs) and Contact Engineering

The development of next-generation integrated circuits requires channel materials that simultaneously have a moderate bandgap for suppressing off-state leakage currents and a low effective mass (m^*^) to achieve high carrier mobility. Theoretical predictions indicate that 2D Bi_2_O_2_S satisfies these stringent requirements. According to calculations performed using the Vienna ab initio Simulation Package, a fully hydrogen-passivated Bi_2_O_2_S monolayer is predicted to exhibit intrinsic electron mobilities ranging from 16,447 to 26,699 cm^2^ V^−1^ s^−1^ at room temperature [29]. The extraordinary transport characteristics of Bi_2_O_2_S are attributed to its electron effective mass (*m*_e_^∗^ ≈ 0.15 *m*_0_), which is lower than that of conventional TMDs such as MoS_2_ (*m*_e_^∗^ ≈ 0.45 *m*_0_) [54].

However, realizing these theoretical properties in practical devices requires mitigating the high contact resistance inherently occurring at 2D semiconductor-metal interfaces [55,56,57]. The nature of electrode materials determines the Fermi-level pinning (FLP) behaviors in Bi_2_O_2_S. In a previous study, calculations predicted that Pd and Pt form p-type Schottky contacts with Schottky barrier heights of 0.54 and 0.99 eV, respectively, owing to strong normal FLP induced by metal-induced gap states [29]. In contrast, incorporating low-work-function metals, such as Ti and Sc, causes an anomalous FLP behavior. Local interface charge redistribution generates a strong dipolar electric field, effectively raising the Fermi level toward the conduction band minimum of Bi_2_O_2_S, thereby forming an ideal n-type Ohmic contact that facilitates efficient carrier injection.

**Figure 8:**
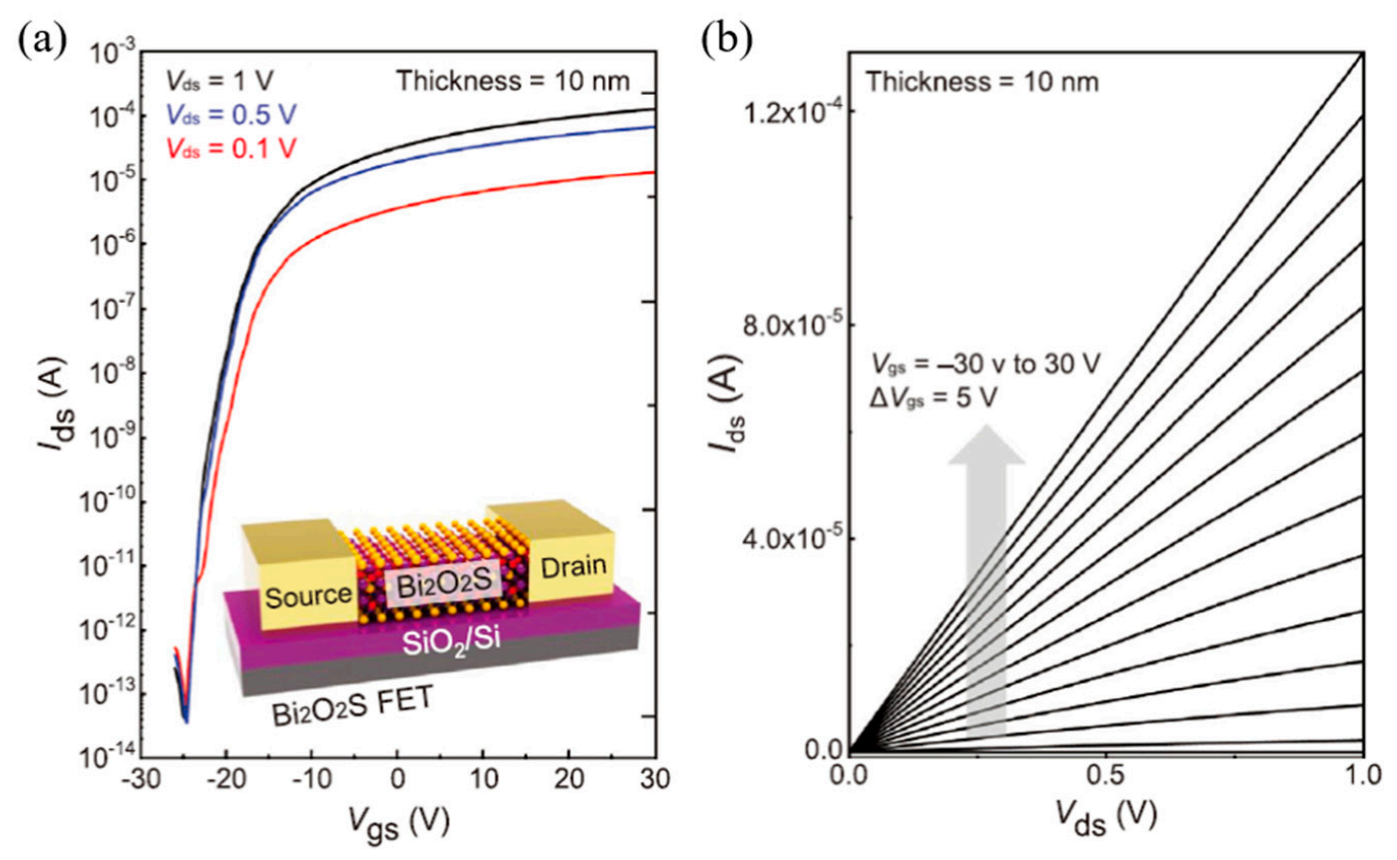
(**a**) Transfer curves of MOCVD-synthesized Bi_2_O_2_S FETs at drain voltages ranging from 0.1 to 1 V. (**b**) Output curves of multilayer Bi_2_O_2_S FETs measured at various back-gate voltages. Reproduced with permission [23].

Recently, FETs were demonstrated utilizing Bi_2_O_2_S channels synthesized via low-temperature MOCVD (Figure 8) [57]. In this study, Ti/Au electrodes were employed to reduce the Schottky barrier height. For example, back-gated FETs fabricated from 6 to 24 nm-thick Bi_2_O_2_S channels exhibited n-type transfer characteristics. With increasing channel thickness, the field-effect mobility increased from 66 to 155 cm^2^ V^−1^ s^−1^, whereas the on/off current ratio decreased from 10^9^ to 10^7^. The ultrathin channels exhibited a lower field-effect mobility due to extrinsic surface scattering sources induced by polymer residues and atmospheric contaminants, such as oxygen and water. The detrimental effects of these adsorbates were suppressed under vacuum conditions, yielding up to a 4.5-fold enhancement in channel mobility compared to ambient conditions. In contrast, when the channel thickness exceeds 22 nm, the electrostatic gate controllability significantly degrades, leading to ineffective modulation of the channel carrier concentration and a consequent increase in the off-state leakage current.

A top-gate FET incorporating a high-κ dielectric layer was further fabricated to mitigate the adverse effects of unpassivated surfaces and interface scattering. In particular, the Bi_2_O_2_S channel capped with a 50 nm-thick Al_2_O_3_ dielectric layer via atomic layer deposition (ALD) exhibited a maximum field-effect mobility of 227 cm^2^ V^−1^ s^−1^ and an on/off current ratio of 10^7^. However, the deposition of these high-κ dielectrics can induce unintended n-type doping in Bi_2_O_2_S, resulting in a negative threshold-voltage shift. Furthermore, such an encapsulated Bi_2_O_2_S FET maintains its electrical characteristics with negligible degradation, even after months of ambient exposure.

### 5.2. Broadband Photodetectors Covering the UV Region to the NIR Region

Bi_2_O_2_S has been investigated as a broadband photoactive semiconductor owing to its optical absorption abilities extending from the UV to the NIR spectral regions. Additionally, bulk and few-layer Bi_2_O_2_S are predicted to exhibit optical absorption coefficients of 1.7 cm^−1^ higher than those of commercial Si and GaAs [28,58]. Early studies on Bi_2_O_2_S-based photodetectors utilized ultrathin nanosheets synthesized via hydrothermal synthesis or mild wet-chemical methods to form lateral metal–semiconductor–metal (MSM) structures [38,53]. Li et al. demonstrated that hydrothermally synthesized Bi_2_O_2_S photodetectors exhibited a photoresponsivity (*R*) of 0.059 A W^−1^ [38]. This optoelectronic performance was further enhanced through structural optimization. For example, Chitara et al. fabricated Bi_2_O_2_S photodetectors exhibiting excellent performance under 785 nm illumination, achieving a high *R* of 4.0 A W^−1^ and an external quantum efficiency of 630% [53]. The effective separation of photogenerated electron–hole pairs in these devices results in a normalized photocurrent-to-dark-current ratio of 1.3 × 10^10^ W^−1^ and a response time of 100 ms.

**Figure 9:**
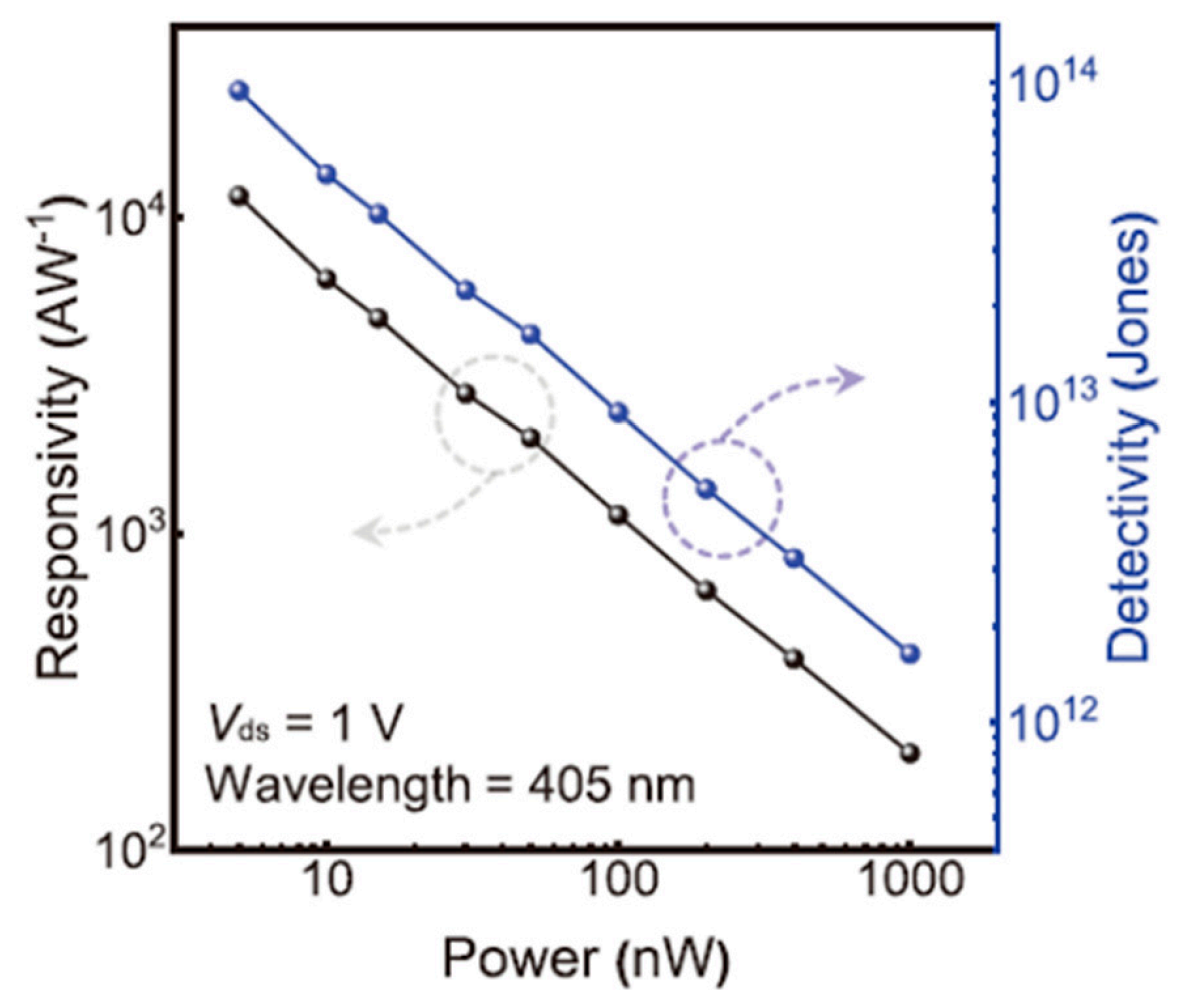
Incident power-dependent photoresponsivity and specific detectivity of an MOCVD-synthesized Bi_2_O_2_S device. Reproduced with permission [23].

To further overcome the limited optical cross-section of atomically thin 2D flakes, morphological engineering has been implemented. Specifically, the light-trapping capabilities of Bi_2_O_2_S were significantly enhanced by employing 3D hierarchical microflowers formed through the self-assembly of 2D ultrathin nanosheets [40]. The spatial gaps within the flower-shaped 3D structure promote multiple internal reflections of incident light, thereby increasing the photon absorption capability. Consequently, these hierarchical Bi_2_O_2_S photodetectors achieved an improved specific detectivity (D^*^) of 9.96 × 10^10^ Jones and a response time of 27.74 ms under 850 nm irradiation. The outstanding advancement in the optoelectronic performance of Bi_2_O_2_S has been recently achieved through low-temperature MOCVD techniques (Figure 9) [23]. These MOCVD-grown crystals exhibit rise and decay times of 6.3 and 4.0 ms, respectively. Furthermore, the maximum *R* and D^*^ approach 11,577 A W^−1^ and 10^14^ Jones, respectively, outperforming commercial Si-based photodiodes, such as the Hamamatsu S1133 (*R*_Si_ = 2.75 × 10^2^ mA W^−1^, *D*_Si_^*^ = 3.8 × 10^12^ Jones at 525 nm) [59]. However, unlike commercial photodiodes, a constant external bias must be applied to extract photogenerated carriers efficiently. Therefore, to realize practical Bi_2_O_2_S-based photodetectors, these characteristics must be achieved in self-powered or low-power operating modes.

### 5.3. Photoelectrochemical and Self-Powered Devices

**Figure 10:**
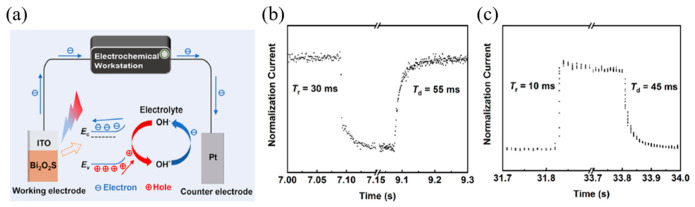
(**a**) Schematic illustration of the operational mechanism for a Bi_2_O_2_S-based PEC photodetector. (**b**) Photoresponse of a self-powered device during light on/off cycles (365 nm) in 1.0 M KOH. (**c**) Photoresponse characteristics of the device operated at an applied bias of 0.6 V under identical illumination and electrolyte conditions. Reproduced with permission [37].

Conventional optoelectronic devices, such as FET configurations, usually require a continuous external bias voltage to separate photogenerated carriers, inherently leading to high static power consumption. In contrast, in a photoelectrochemical configuration, carrier separation is spontaneously facilitated by band bending at the semiconductor/electrolyte interface under zero external bias (Figure 10a) [37]. Thermodynamic equilibration between the Fermi level of the semiconductor and the redox potential of the electrolyte induces a built-in electric field that enables the spontaneous separation and migration of photogenerated charge carriers.

In alkaline or neutral liquid electrolytes, such as KOH or Na_2_SO_4_, Bi_2_O_2_S exhibited outstanding broadband detection capabilities ranging from the UV (365 nm) to NIR (850 nm) [37]. At 365 nm, the zero-bias device exhibits an *R* of 2.40 mA W^−1^ and a D^*^ of 6.06 × 10^9^ Jones, with rise and decay times of 30 and 50 ms, respectively (Figure 10b). When a bias of 0.6 V is applied, charge extraction becomes more efficient: the *R* and D^*^ increase to 13.0 mA W^−1^ and 2.34 × 10^10^ Jones, respectively, while the rise and decay times decrease to 10 and 45 ms, respectively (Figure 10c). Variations in the ionic concentration and the specific chemical nature of the electrolyte alter the interfacial charge-transfer kinetics and the resulting photoresponse characteristics. For example, increasing the KOH concentration increases the photocurrent, while Na_2_SO_4_ produces a low photoresponse despite exhibiting a lower interfacial resistance than dilute KOH, indicating that redox kinetics and anion adsorption at the Bi_2_O_2_S surface must be collectively considered when interpreting PEC performance.

Despite these remarkable optoelectronic properties, conventional PEC devices face practical bottlenecks, particularly liquid electrolyte leakage and rigid packaging constraints, which substantially hinder their application in next-generation wearable technologies [60]. To address these limitations, a PET-based flexible detector was recently fabricated using Bi_2_O_2_S nanosheets as the photoactive layer and a DI water/PVA solid-state electrolyte as the ion-conducting medium [61]. The device produced a photocurrent density of 0.55 μA cm^−2^ at 0 V under 75 mW cm^−2^ illumination and exhibited an *R* of up to 1.02 × 10^−2^ mA W^−1^ with a response time of 45 ms. Structural fatigue tests revealed that the device maintained over 91% of its original photocurrent density even under a bending angle of 30°. The device further showed negligible performance degradation even after repeating thousands of on/off operation cycles.

Table 2 presents the performance metrics of the Bi_2_O_2_S photodetectors.

## 6. Conclusions and Future Perspectives

### 6.1. Summary of Current Progress

Research on Bi_2_O_2_S has transitioned from fundamental synthesis and theoretical predictions toward early device demonstrations. Bi_2_O_2_S flakes were initially synthesized through solution processes, including room-temperature wet-chemical synthesis and organic-ion-template-guided hydrothermal routes [26,38]. These techniques facilitated the preparation of freestanding ultrathin nanosheets and self-assembled 3D hierarchical microflowers, enabling exploration of fundamental physical properties [40]. More recently, the synthesis of highly crystalline Bi_2_O_2_S flakes has been achieved via low-temperature MOCVD [23,25]. Advances in vapor-phase processing have facilitated the large-scale growth of phase-pure single crystals within the Bi_2_O_2_S ternary compounds, thus mitigating the inherent thermodynamic instability and ensuring compatibility with the thermal budget of the BEOL process. The orthorhombic lattice of Bi_2_O_2_S exhibits distinct polarization behaviors. The localized inversion symmetry breaking within the [Bi_2_O_2_]^2+^ layers induces room-temperature 2D ferroelectricity and antiferroelectricity [25,26]. Furthermore, precise defect engineering, such as OV formation and Se substitutions, has emerged as a viable pathway to modulate the electronic properties of Bi_2_O_2_S [24]. These strategies introduce mid-gap states, narrow the optical bandgap, and accelerate photocarrier relaxation dynamics into the sub-picosecond regime. Bi_2_O_2_S FETs fabricated using nanoplates grown by the MOCVD method demonstrated robust electronic transport, achieving a field-effect mobility of up to 227 cm^2^ V^−1^ s^−1^ and on/off current ratios exceeding 10^9^ [23]. In addition, PECs composed of Bi_2_O_2_S and a solid electrolyte provide broadband, self-powered properties while maintaining high mechanical flexibility [61].

### 6.2. Challenges and Future Directions

Despite recent progress, the primary challenge in Bi_2_O_2_S research is to implement a scalable synthesis method and develop a silicon-compatible process. Recent MOCVD studies have demonstrated that highly crystalline Bi_2_O_2_S flakes can be synthesized at approximately 400–430 °C, a processing window compatible with the BEOL thermal budget [23]. However, lateral coalescence of individual flakes into continuous single-crystal films remains unresolved. Therefore, future research should focus on nucleation-density control, lateral coalescence kinetics, and spatial thickness uniformity. The second challenge is robust control of charge-carrier polarity. Most experimentally implemented Bi_2_O_2_S FETs exhibit intrinsic n-type transport, where donor-type native defects typically complicate or compensate p-type doping. To realize complementary logic circuits, the preparation of p-type channels with performance comparable to that of n-type channels is essential. Hence, stable, controllable p-type doping methods should be developed. The third bottleneck is interfacial and surface stability. Dielectric deposition, lithography residues, oxygen and water adsorption, and post-growth thermal annealing can alter the channel, degrading the intrinsic carrier mobility. Concepts previously developed for Bi_2_O_2_Se, such as native-oxide gate stacks or monolithic 3D gate-all-around integration, remain highly valuable to Bi_2_O_2_S [16]. Therefore, discovering lattice-matched, high-κ native oxides suitable for Bi_2_O_2_S represents a highly viable strategy. Finally, while the room-temperature ferroelectricity of Bi_2_O_2_S is scientifically significant, further research is required on domain retention, fatigue resistance, switching statistics, and array-level reproducibility. By leveraging these ferroelectric properties, Bi_2_O_2_S can also be utilized in next-generation memory or neuromorphic electronics.

## Figures and Tables

**Table 1:** Comparison of synthesis methods for Bi_2_O_2_S.

Synthesis Method	Precursor	Process Temperature	Process Time	Domain Size	Thickness	Refs.
Hydrothermal	Bi(NO_3_)_3_⋅5H_2_O, SC(NH_2_)_2_	120 °C	3 h	2 μm	12–16 nm	[40]
Hydrothermal	Bi(NO_3_)_3_⋅5H_2_O, SC(NH_2_)_2_	200 °C	30 min	600 nm	55 nm	[24]
Hydrothermal	Bi(NO_3_)_3_⋅5H_2_O, SC(NH_2_)_2_	200 °C	3 days	300 nm	-	[27]
Hydrothermal	C_6_H_13_BiN_2_O_7_⋅H_2_O, SC(NH_2_)_2_	200 °C	Several hours	2 μm	4.6 nm	[38]
Wet chemical	Bi(NO_3_)_3_⋅5H_2_O, SC(NH_2_)_2_	Room temperature	10 h	20–40 nm	2–4 nm	[37]
Wet chemical	Bi(NO_3_)_3_⋅5H_2_O, SC(NH_2_)_2_	Room temperature	overnight	80–100 nm	2–3 nm	[53]
Wet chemical	Bi(NO_3_)_3_⋅5H_2_O, SC(NH_2_)_2_	Room temperature	10–15 min	150–300 nm	2 nm	[26]
MOCVD	Bi(Ph)_3_, (CH_3_CH_2_)_2_S, O_2_	400–430 °C	3–6 h	20 μm	6–24 nm	[23]
PLD	Bi_2_O_2_S	450 °C	-	-	25–200 nm	[25]

**Table 2:** Performance comparisons of Bi_2_O_2_S-based photodetectors.

Synthesis Method	Wavelength (nm)	Operation Voltage (V)	Responsivity (mA/W)	Detectivity (Jones)	Response Time (ms)	Refs.
Hydrothermal	850	0 V	9.5	99.96 × 10^10^	27.74	[40]
Hydrothermal	532	10 V	59	4.97 × 10^9^	81.9	[38]
Wet chemical	365 365	0 0.6	2.4 13	6.06 × 10^9^ 2.34 × 10^10^	30 10	[37]
Wet chemical	785	5	4.0 × 10^3^	-	100	[53]
Wet chemical	White	0	1.0 × 10^−2^	-	45	[61]
MOCVD	405	1	1.2 × 10^7^	10^14^	4	[23]
PLD	620	-	60	3.45 × 10^11^	200	[25]

## Data Availability

No new data were created or analyzed in this study. Data sharing is not applicable to this article.
